# Investigation of Temperature Cycling with Coupled Vessels for Efficient Deracemization of NMPA

**DOI:** 10.1021/acs.cgd.2c01138

**Published:** 2023-07-10

**Authors:** Ghufran ur Rehman, Thomas Vetter, Philip A. Martin

**Affiliations:** Department of Chemical Engineering, University of Manchester, Manchester M13 9PL, U.K.

## Abstract

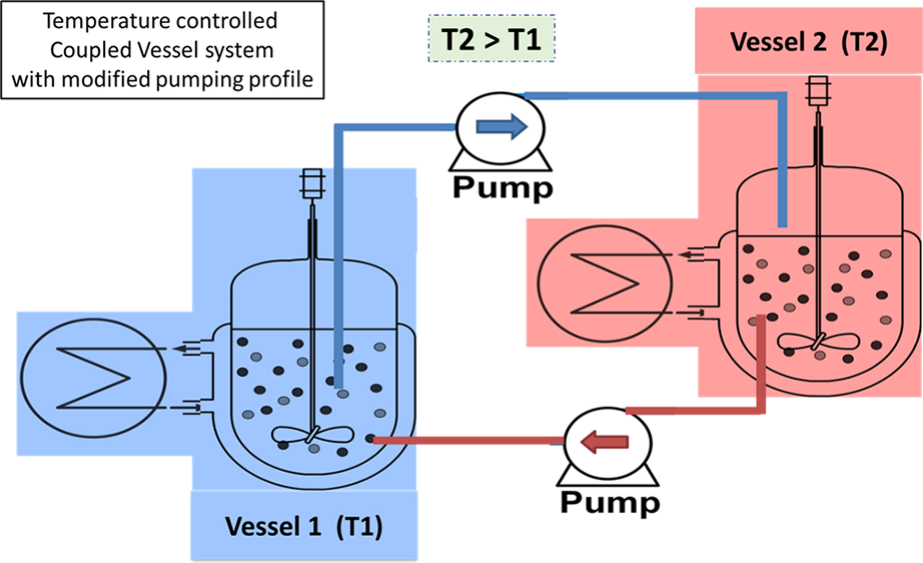

Chiral compounds can exist as pairs of nonsuperimposable stereoisomers (enantiomers) possessing the same physical properties but interacting differently with biological systems. This makes them interesting materials to be explored by the pharmaceutical and food industries. In this study, to obtain pure enantiomers from their conglomerates, a method that involves using a two-vessel system for deracemization of *N*-(2-methylbenzylidene) phenylglycine amide (NMPA) was developed. In this method, a suspension was transferred with a pulsating pumping profile between two inter-connected stirred vessels that were set at constant temperatures. As the suspension was exposed to more rapid changes in temperature, it resulted in the speeding up of the process and thus enhancing productivity in comparison to a single vessel system. The results confirmed successful deracemization of NMPA. A modified pumping profile and tubing design eliminated the issue of clogging of the transfer tubes and ensured effective suspension transfer for longer durations. Operating parameters, such as initial enantiomeric excess, vessel residence time, and suspension density were also investigated. In this method, optimization of residence time was necessary to enhance the efficiency of the process further. Results confirmed that this methodology has the potential to be more adaptable and scalable as it involved no mechanical attrition.

## Abstract 2

The Temperature Cycling Coupled Batch system (TCCB) confirmed an improved alternative setup for deracemization via temperature cycling. These experiments showed successful resolution of an NMPA model compound and its capability of reaching high purity in a shorter process time as compared to a single vessel setup. A modified pumping profile increased the efficiency of the process.

## Introduction

The preparation of pure enantiomers of chiral molecules is important for a range of applications including pharmaceutical manufacturing. Chiral compounds are commonly produced via chemical synthesis methods as an equimolar mixture of two enantiomers (racemic mixture) so that an additional step is therefore necessary to remove the undesired component. Crystallization is one process that has been used to separate or purify different enantiomers. Enantiomers can crystallize either as a racemic compound (enantiomers in a structured array with an equal proportion in a single crystal), a conglomerate (physical equimolar mixture of enantiomers in two different crystals), or a solid solution (*<*2%).^1,2^ Chiral crystallization (chiral resolution) processes are mostly used for resolving conglomerate as the enantiomers are more easily separable. Out of various chiral resolution methods, Preferential Crystallization (PC) and Viedma Ripening (VR) processes provide an effective way to achieve conglomerate separation.^3^ PC is a stereo-selective process which is initiated by adding seed crystals into its racemic supersaturated solution and the desired enantiomer is selectively crystallized. Viedma Ripening (VR) is a process that involves the abrasive grinding of a crystal mixture with a small initial excess of the desired enantiomer under saturated conditions. Viedma used glass beads for grinding a sodium chlorate NaClO_3_ racemic mixture, leading to a pure enantiomer with 100% yield.^4^ VR is the combination of solid-state processes involving a liquid phase racemization reaction, leading to complete deracemization of the solid phase.^4,5^ Experimental deracemization of *N*-(2-methylbenzylidene) phenylglycine amide (NMPA) has also been reported by utilizing an attrition-enhanced deracemization approach.^6^

Several modeling studies have been attempted to investigate the factors affecting the racemization in solution.^7−10^ It was shown through modeling that attrition was not the only factor necessary to complete deracemization. However, it enhanced the rate of the process in terms of faster resolution. Viedma et al., then went on to demonstrate experimentally for the first time that only by heating the suspension without grinding could also lead to complete deracemization.^11^ A suspension of NaClO_3_ crystals was subjected to boiling, resulting in temperature-induced cycles of crystal growth and dissolution that led to chiral purity being achieved in 24 h. The method involved producing a temperature difference of 14 °C between the lower and upper parts of the flask and initiating a dissolution-growth process of the crystals which finally generated a homochiral system. However, mechanisms during boiling made it challenging to understand and control the deracemization process, and a more controllable method of inducing a temperature fluctuation was required.

In 2013, Suwannasang et al. reported^12^ on a deracemization process by programmed temperature cycles involving a heating and cooling ramp separated by a holding time so that equilibrium can be reached at a set temperature. During the heating ramp and hold time, a proportion of crystal suspension was dissolved depending on the solubility and racemization of dissolved crystals present in the liquid phase, whilst in the cooling period, supersaturation of the solution gave rise to growth of the crystals in suspension from the racemized crystals in the liquid phase.^12^ This approach demonstrated a faster (50–90 h duration) completion of the deracemization process as compared to the grinding and boiling mechanisms. A similar mechanism may occur during grinding but with a more localized energy/temperature variation (due to friction), leading to dissolution and growth of crystals. In recent years, extensive research has been carried out on modification of the thermal mechanism, including temperature fluctuation^13−16^ and the use of microwave sources.^17^ The thermal cycling approach was optimized further by applying damped temperature cycles, reducing the cycle amplitude over time.^18^

Suwannasang and Breveglieri et al. reported that a faster cooling rate resulted in a slower ee evolution. The faster cooling rate resulted in nucleation of the unwanted enantiomer that caused a further delay in the process. However, Li et al.^13^ contradicted this claim and reported no effect of the cooling rate on the deracemization completion time. In this case, the negative impact on the overall deracemization time was countered by the reporting that the faster cooling rate resulted in a shorter cycle time, and, in return, a greater number of cycles were processed in a set time period. It was demonstrated that smaller swings led to faster ee evolution.^19,20^ However, Li et al. demonstrated the opposite effect^13^ which may be due to the faster racemization rate at higher temperature swings. This showed that there was a complex interplay of different parameters involved in this process, influencing the overall progress. Studies involved the synthesis of conglomerate compounds that were compatible for the deracemization process.^21^ Model simulations and laboratory experiments were performed in parallel to validate findings and to understand the overall process in more detail. Studies reported by Iggland^22^ and Bodak^9^ on attrition-enhanced and temperature cycling deracemization processes modeled the impact of initial enantiomeric excess on process progress. Both studies showed that higher initial excess results in faster deracemization progress which agreed with experimental work. Interestingly, model simulation on the initial racemic mixture with varied crystal size distribution (one enantiomer having smaller size crystals than the other) resulted in complete deracemization. This provided a good explanation of how a racemic mixture progressed toward obtaining the pure enantiomer as the varied enantiomer crystal size resulted in faster dissolution of one, whereas the other resulted in the initiation of enantiomeric excess from an overall racemic mixture. These sources of asymmetry acted in competing directions, one dominated the other or, conversely, they might equally deracemize, resulting in the suspension remaining as a racemic mixture. The temperature cycling process was critically dependent on the heating and cooling capacity of the control system and the switching between the two. However, upscaling to larger volumes required a substantial heat transfer area for effective heating and cooling cycles, resulting in a longer time required for dissolution/growth of crystals. It was concluded that the primary mechanisms at work in the temperature cycling process were the dissolution and growth cycles, along with enantioselective incorporation. The temperature cycling process appeared to be a suitable candidate for scale-up as compared to Viedma ripening as the only control requirement was temperature. Steendam et al.^16^ attempted to investigate the process for volumes of 1 L and observed that with larger volume vessels, the temperature differences between the suspension and the crystallizer vessel led to nonselective nucleation and agglomeration, resulting in an inhibition of reaching 100 ee%. This issue was countered by use of a dispersing tool that homogenized the suspension by breaking crystals into fine particles and preventing agglomeration. This was important for scaling-up temperature cycling to an industrial level.

Suwannasang et al.^19^ further developed the temperature cycling approach by using coupled mixed-suspension vessels. The approach involves applying the cycling of the suspension continuously between two vessels that are held at different temperatures (high and low) based on solubility differences.^19^ This allows faster resolution due to a shorter cycle time, equivalent to one residence time of the system. Suwannasang reported complete deracemization within 12 h. In the two-vessel system, the dominant mechanism occurring in the hot vessel is dissolution of the enriched suspension into the liquid phase and racemization of the dissolved crystals in the liquid phase whilst growth and agglomeration of enriched crystals in the solid phase occurs in the cold vessel. In the solid phase, the desired enantiomer is slightly enriched, giving more surface for the enantiomer to grow. As a result of growth, it leads to consumption from the saturated solution. The deficiency of the desired enantiomer is compensated by the suspension transfer from the hot vessel, which already has an excess of the desired enantiomer obtained by the racemization reaction in the liquid phase. This transfer between two vessels gradually results in deracemization into a product of pure enantiomer and is complete when all the dissolved suspension was racemized and grown in the solid phase. Compared to a single vessel, the two-vessel approach requires additional apparatus for temperature control and pumps for suspension transfer, but only isothermal control for both vessels is required. This can prove to be more cost effective in terms of power and enantiopure crystals being obtained in a single-step temperature operation.

Whilst Swannasang’s twin-vessel concept was novel with the basic principles outlined, there was little in-depth analysis of the influence of the process variables. In this work, we aim to study these process variables in more detail such as the influence of modified pumping profiles, the effect of varying the initial ee%, the residence time, and the initial suspension wt % for the two-vessel system. This work also aims to link the previous work reported by Breveglieri et al. on NMPA temperature cycling deracemization^20,23^ single vessel system as NMPA has not been studied in a two-vessel system.

The structure of the article is as follows: first, we explain the design and working of the temperature cycling coupled vessels setup and its modified pumping mechanism, followed by initial experiments and results of a small-scale vessel test via Crystal16 equipment. (These were performed beforehand to find suitable initial parameters for the main process.) Second, the results acquired from Crystal16 experiments are discussed and applied to the coupled vessel setup. For the two-vessel system, a modified pumping profile, variation of initial ee%, variation in residence time, and variation in suspension wt % are investigated, and a comparative analysis carried out for the coupled vessel influences the enantiomeric excess (ee) as a function of process time.

## Experimental Section

### Materials

*N*-(2-Methylbenzylidene)-phenylglycine amide (NMPA) was used in a solvent mixture of 95/5 (w/w) iso-propanol (IPA) and acetonitrile (ACN) to prepare a saturated solution with solubility data obtained from recent research work.^20^ The saturated solution was stirred overnight at 25 °C and was then filtered using vacuum filtration.

The deracemization reaction of NMPA was performed in the presence of the racemization agent 1,8-diazabicyclo[5.4.0]undec-7-ene (DBU) that was reported in previous studies.^6,22^ Solvents and racemization agents of purity >99% were purchased from Sigma Aldrich. NMPA conglomerates were synthesized according to the three-step method described by Iggland et al. and NMPA pure *S*-enantiomer obtained by deracemization reaction as reported.^20,22,23^ To make NMPA of 1.5 wt % suspension density, the NMPA racemic mixture was used. The initial enantiomeric excess (ee_o_) was determined using the following equation:
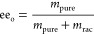
1*m*_pure_ is the weight of pure enantiomer, while *m*_rac_ represents the weight of the racemic mixture, which was later added to make suspensions in saturated solutions. The calculated amount was then added in the weighed out racemic mixture of 1.5 wt % suspension density.

### Experiment Setup and Procedure

**Figure 1:**
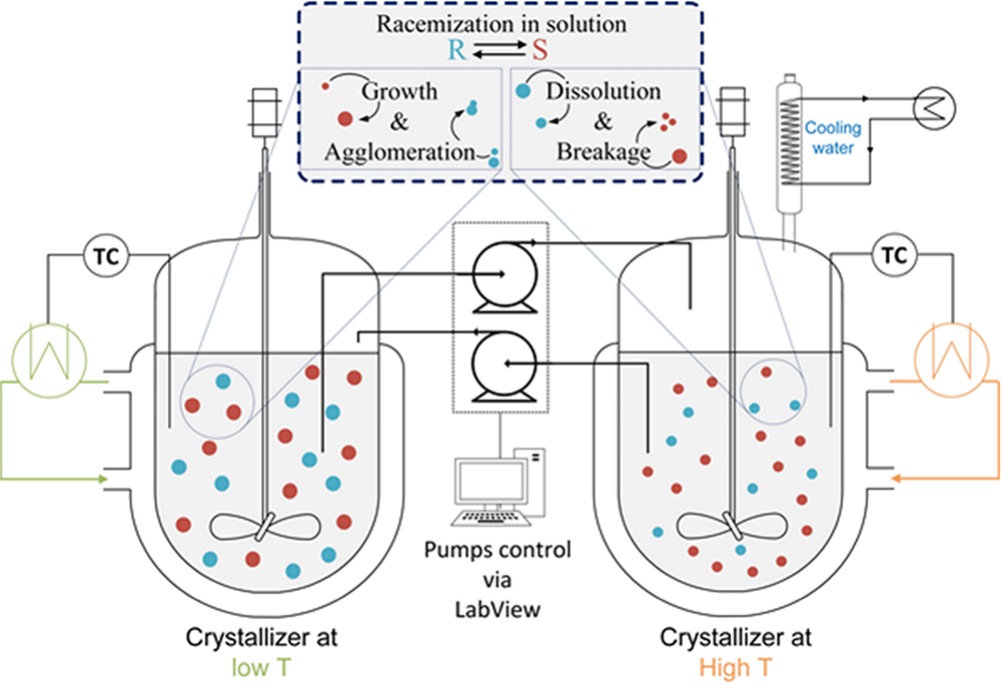
Schematic of two-vessel crystallizer mixed suspension setup connected via computer-programmed pumps and temperature-controlled thermostats. Growth and agglomeration are dominant in the cold vessel and dissolution and racemization in the high *T* vessel.

**Figure 2:**
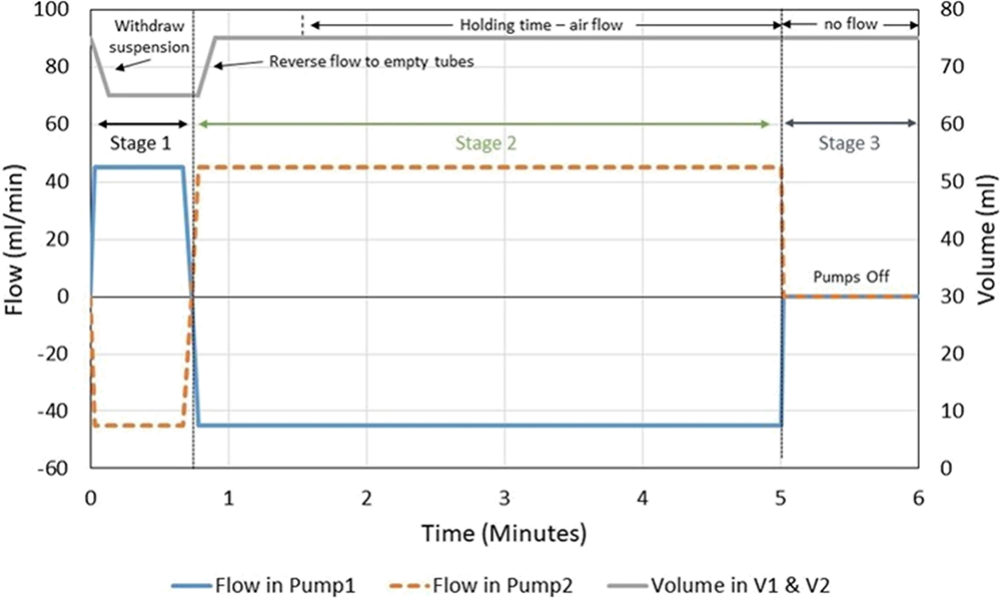
Pump flow and volume profile of crystallizer vessels during temperature cycling coupled-vessel experiments. A 3 stage pumping profile is shown and its effect on the volume of vessels. Stage 1 is initiated by withdrawing suspension from both vessels. Stage 2 reverses the flow to empty the tubes followed by a holding time that was integrated with the next step stage 3, where the pump was stopped for 1 min.

Figure 1 shows the experimental setup that consists of two temperature-controlled jacketed stirred vessels that were interconnected via masterflex tubing and pumps. Vessel temperature was maintained by using thermocouple-controlled water baths. This approach was first proposed by Suwannasang et al.^19^ by continuous transfer of suspension between two vessels. In this study, a working volume of 75 mL was maintained in both vessels. A total volume of 150 mL was utilized for the entire process. Programmable ColeParmer peristaltic pumps were used for transferring the crystal suspension between the two vessels, with a relatively low constant flowrate (25 mL/min). Gentle peristaltic pumping ensured smooth flow and avoided breakage of crystals. The tube volume connecting the two vessels was measured to be around 20–30 mL, depending on the internal diameter and the length of the tube. In the previous studies, it has been shown that a two-vessel system produced larger crystals than conventional single vessel temperature cycling processes^20^ due to the fact that in a cold vessel, crystals grow continuously. To address this issue, a study by Maggioni et al.^24^ reported the use of pumping through a homogenizer and adding a surfactant to the suspension. However, this may impact the process mechanism as it will require an additional separation step to remove the surfactant from the final product. As an alternative to a dispersing tool, a modified pumping profile was used for this method, as a similar approach was proposed by Köllges et al.^25^ A modified pumping profile with a three-stage mechanism is shown in Figure 2. Stage 1 transfers an equally calibrated amount of suspension between the two vessels (20% of total vessel volume) followed by stage 2, reversing the flow and emptying the suspension in the tube. Stage 3 is the holding time to attain the required residence time of the vessel. The inlet and outlet tube openings were fixed at a position inside the vessels to ensure that the volume was kept constant (as set) throughout the prolonged deracemization process. Reverse flow was extended further which prevented blockage of the tubes, as shown in Figure 2. The residence time of the coupled vessels was defined by the total residence time of one pumping cycle (*τ*) [s]. It is the sum of residence times during stages 1, 2, and 3 of the pumping profile. The following equation for the residence time is used during the flow between the transfer tubes during stages 1 and 2.
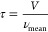
2
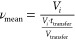
3where *ν*_mean_ [mL s^–1^] is the mean volumetric flow for the specific stage of the cycle, *V*_transfer_ [mL] is the volume of suspension on which flow rate is acted upon for time *t*_transfer_ [s], while *V_i_* [mL] is the volume of each vessel (where, *i* = 1, 2). When the tubes are being emptied, *V*_transfer_ is equal to the volume of the connecting tubes. During normal pumping, this term does not include the volume of the tubes, as the suspension retained in the tubes was not fully transferred. For stage 3, as it encounters no flow, the total time was added to the total residence time of the vessel.

Crystal16 (Multiple Reactor Crystallizer system with 16 crystallizers, 2 mL volume each) experiments were performed beforehand for selection of suitable parameters as a starting point for the investigation. Later, single vessel up-scaled temperature cycling experiments were carried out for comparison with the work done by Breveglieri et al.^20^

#### Procedure: Crystal16 TC Experiments

Temperature cycle test experiments were performed in Crystal16 Equipment. Each 2 mL vial consisted of weighed suspension of NMPA (enriched with *S* enantiomer for obtaining initial excess) in its racemic mixture saturated solution. The added suspension was measured according to the temperature-dependent solubility and temperature cycle set points. Parameters are listed in Table 1. Each cycle consists of low-*T* and high-*T* limits (shown as *T*_1_ and *T*_2_) followed by a holding time in between. Heating and cooling rates were set to 1.33 °C per minute. Each experiment went through a series of cycles until reaching near the end point of the deracemization process.

**Table 1:** Crystal16 Experiments—Values of the Investigated Operating Parameters and Results

exp. TC	susp, (wt %)	initial ee_o_%	*T*_1_ (°C)	*T*_2_ (°C)	Δ*T* (°C)	holding time (min)	*T*-cycle time (min)	ee%	*t*_total_ (h)
1	1.5	10	25	30	5	10	28	75%	31
2	1.5	10	25	30	5	10	28	76%	31
3	1.5	16	25	30	5	5	18	82%	28
4	1.5	12	25	30	5	5	18	84%	28
5	1.5	15	25	33	8	10	32	97%	48
6	1.5	18	25	33	8	10	32	99%	48
7	1.5	18	25	33	8	5	22	98%	26
8	1.5	15	25	33	8	5	22	98%	26

#### Coupled-Vessel Experiments

A saturated solution of the NMPA conglomerate was prepared in a solvent mixture of IPA/ACN according to the solubility data measured gravimetrically in recent research work.^20^ A scheme of the proposed setup is given in Figure 1. A saturated solution of NMPA was introduced in vessels *V*_1_ and *V*_2_ and the desired temperature of both vessels (cold and hot vessel) was set according to the calculations in the pre-experiment plan shown in Table 2 as *T*_1_ for *V*_1_ and *T*_2_ for *V*_2_, respectively. Care was taken in the selection of the high *T* limit in order to prevent dissolution of all suspension crystals as this will result in no growth of the desired enantiomer and an increased likelihood of nucleation of the unwanted enantiomer during the cooling cycles. For experiments with the suspension with enantiomer enrichment, this was prepared by wet grinding of racemic NMPA with the chiral (*S*)-NMPA enantiomer in order to minimize variability between experiments and to attain consistent initial enantiomeric excess. Measured wt % suspension, as shown in Table 2, was introduced in equal proportions into the two vessels with NMPA saturated solution. Once the suspension was well mixed with stirring, the first solid sample was taken at *t* = 0, at *T*_min_ set temperature using the manual sampling method described in the HPLC analysis section. Once the set *T* was achieved, DBU 3.85 μL/g was added and then the pumping profile was initiated by means of a customized LabView program. Sampling was taken after 30 min to 1 h intervals from the cold vessel. Experiments were performed until an ee of >90% was achieved in the cold vessel. Each set of parameters (initial excess, residence time, flow rate, suspension density, and vessel volume) was repeated and investigated at least twice for reproducibility evaluation. The desired crystals were grown in the cold vessel and the racemization of the dissolved suspension occurred in the hot vessel. The modified pumping profile was tested and modified step by step to ensure continuous mixing of suspension whilst maintaining the same residence time of both cold and hot vessels (τ_1_ and τ_2_). At low mean residence times in the two vessels, a fluid element experienced (on average) a more rapid change in temperature in comparison to a batch, which was thought to be beneficial for speeding up the process. The solution volume of both vessels was kept at approximately 75 mL. The volumes should not be much smaller because of the need to ensure reasonable residence times (Figure 2) in the vessels at the flow rates where the particles in the vessels can be transported. During cross-suspension transfer between hot and cold vessels (pumping stage 1), the effect on vessel temperatures at stage 1 flow was around *T*_1_ = 25 °C ± 0.5 °C and *T*_2_ = 34 °C ± 1.2 °C.

**Table 2:** TCCB Experiments—Values of the Investigated Operating Parameters and Results

exp. TCCB	susp (wt %)	initial ee_o_%	*V*_1_ (mL)	*T*_1_ (°C)	*V*_2_ (mL)	*T*_2_ (°C)	residence time τ_1_/τ_2_ = τ (min^*–*1^)	ee > 90%*t*_total_ (h)
01	1.5	20	70	25	40	32	2.67/1.3	
02	0.5	16	70	25	40	32	2.67/1.3	
03	1.5	32	75	25	75	34	9/9	9
03a	1.5	33	75	25	75	34	9/9	8.5
04	1.5	12	75	25	75	34	8.7/8.7	24
05	1.5	22	73	25	77	34	9/9	13
06	1.5	32	75	25	75	34	9/9	8.5
07	1.5	22	75	25	75	34	3.5/3.5	10
08	1.5	23	75	25	75	34	6.05/6.05	7.8
09	1.5	24	75	25	75	34	7.5/7.5	9
10	0.5	40	75	25	75	34	7.5/7.5	

### HPLC Analysis

HPLC measurements to monitor process evolution in terms of enantiomeric excess were conducted on a Thermo Scientific UltiMate 3000 HPLC series equipped with a quaternary pump and a UV–Vis detector. They were carried out in normal phase setting. Measurements of 10 μL injections were carried out at 213 nm on a Daicel CHIRALCEL OJ-H HPLC Analytical Chiral Column, 5 μm, ID 4.6 mm × L 250 mm. For the mobile phase, a 60:40 (v/v) mixture of *n*-hexane (HPLC grade, Fisher Scientific) and iso-propanol IPA (HPLC grade, Fisher Scientific) was used with a flow rate of 1 mL/min. Retention times were found to be 6.5 and 9.0 min for *S*-NMPA and *R*-NMPA, respectively. The enantiomeric excess ee was calculated by analyzing the peak area for each enantiomer.

#### Sampling

Two samples of <0.1 mL were taken after each hour interval using a pipette. The first sample was taken before the addition of racemizing agent (DBU) for determining the starting point of the process. Two solid samples were collected by vacuum filtration (MS PTFE Membrane Filter 0.45 μm). When the sample was completely dry and the solvent evaporated it was then washed with 1–2 drops of methyl *tert*-butyl ether (TBM, Sigma Aldrich) to remove traces of DBU and solvents followed by placing the crystals carefully in separate vials and allowing them to dry (for 30 min). After drying, IPA was used as a solvent to dissolve the crystals which were also sonicated for 5 min to dissolve all the sample crystals. The solution was then transferred to the HPLC vials by a filter syringe for analysis. The following equation was used for calculating enantiomeric excess from the HPLC peak.
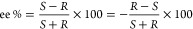
4where *S* and *R* are the peak areas of enantiomer measured by HPLC at 6.5 and 9 min retention time, respectively. The degree of excess of one enantiomer over the other in a racemic mixture is expressed as the enantiomeric excess (ee).

## Results and Discussion

### Crystal16 Single Vessel Temperature Cycling

Initial trial experiments were performed in a single vessel (Crystal16 setup) to examine the parameters for the two-vessel process. It was crucial to investigate initial parameters, such as temperature set-points (Δ*T* (*T* high – *T* low)) and residence time (in a single vessel, it is regarded as the holding time). For this purpose, Crystal16 experiments were performed to determine the suitable Δ*T* and holding time as a reference point. Table 1 shows the values of initial parameters investigated. In all experiments, the solid suspension density (1.5 wt %), racemization agent DBU concentration (3.85 μL/g), and *T* low-saturated solution temperature were kept constant. Three repetitions were performed to check on the reproducibility of results.

**Figure 3:**
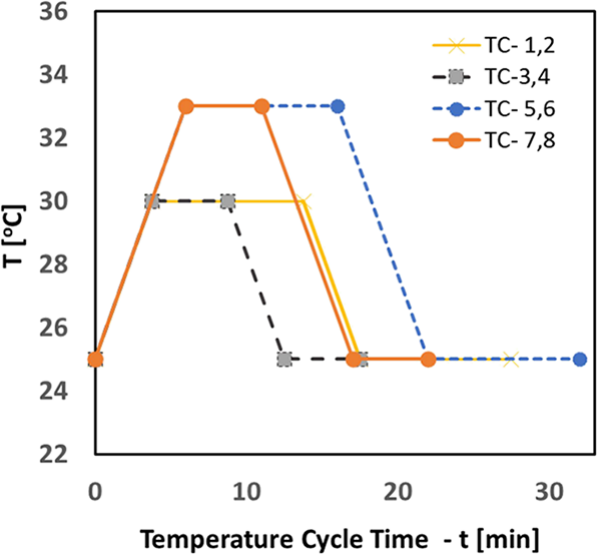
Crystal16 Experiments TC-1 to TC-8 heating and cooling cycle experiment profile, representing operating parameters applied values shown in Table 1. *T* represents temperature in °C and *t* represents temperature cycle time in minutes.

Initial enantiomeric excess (ee_o_) was maintained in the range of 10–18%. *T*_1_ and *T*_2_ were the minimum and maximum temperature points for the cycling process with Δ*T* as the difference between these two temperature set points. The holding time, as shown in Figure 3, is required after the heating and cooling temperature set points are reached to allow time for growth, dissolution, and racemization of crystals. Heating and cooling rates were kept to 1.33 °C/min. The holding time was helpful in evaluating the mean residence time required for the two-vessel system, which included an approximation of holding time and the time taken during the heating and cooling cycle in a single-vessel process.

**Figure 4:**
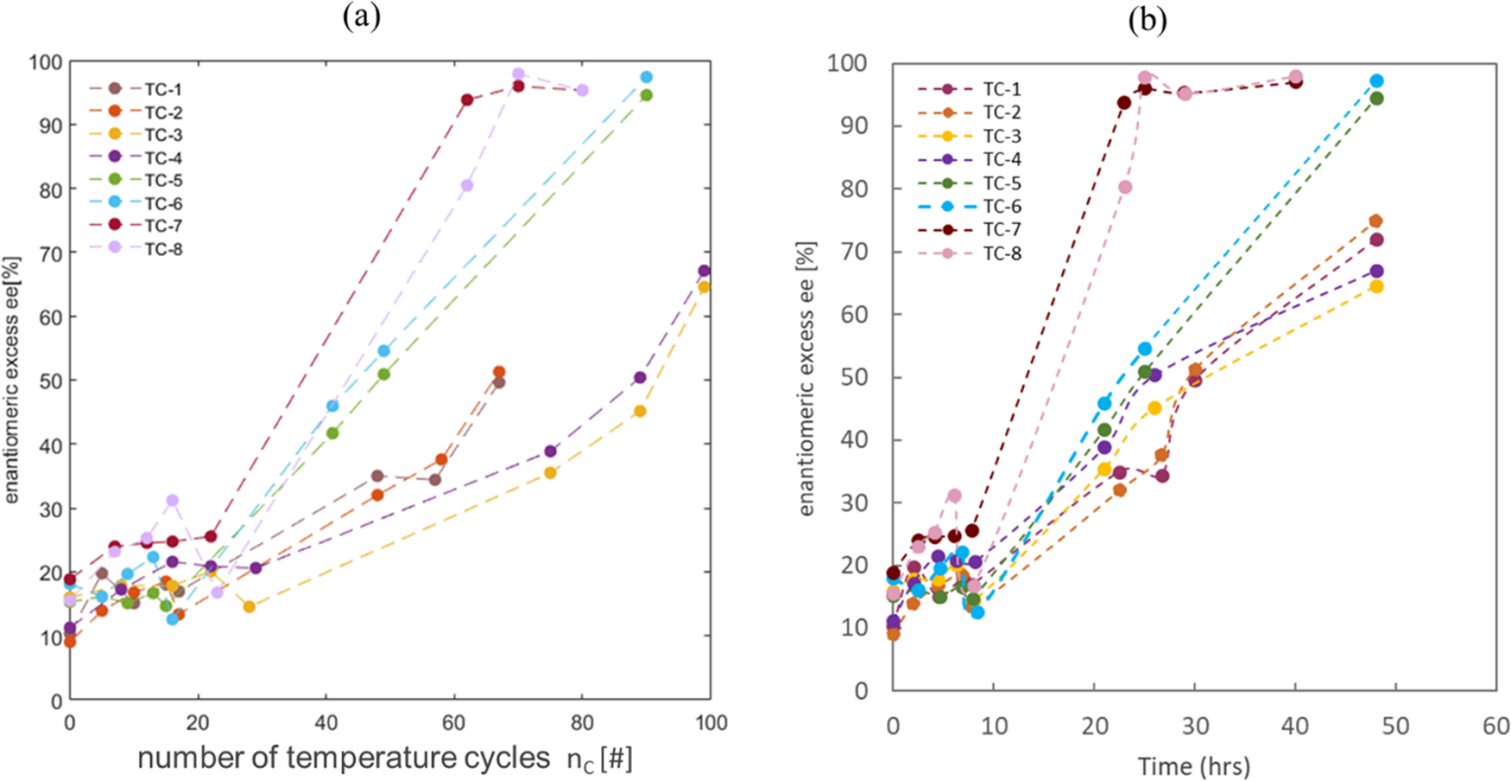
Crystal16 experiments results: TC-1 to TC-8 representing operating parameters applied values given in Table 1; (a) HPLC results analysis depicting evolution of enantiomeric excess with reference to the number of temperature cycles *n*_c_. (b) Enantiomeric excess evolution against overall time in hours.

The results TC5–TC8 indicated that a Δ*T* of 8 °C followed a faster ee% evolution irrespective of the slight variation in initial excess (15 and 18%). A holding time of 5 min with Δ*T* of 8 °C depicted the fastest result by reaching close to 100 ee% in the fewest number of cycles and time shown in Figure 4. Also, a lower holding time resulted in increased efficiency of the process with less time required (26 h) to achieve complete deracemization of enantiomers, as shown in Table 1. Other parameters TC-1 to TC-4 indicated that it took a longer time and an increased number of cycles to reach complete deracemization and some of them never reached completion end point even after 48 h of temperature cycling. This helped understand and identify the most favorable parameters to investigate initially with the coupled vessel system.

### Temperature Cycling in Coupled Batch

Table 2 shows a summary of all two-vessel experiments carried out including the selected parameters for investigation. For the two-vessel system, the modified pumping profile (that includes reverse flow) for variable residence time τ, as shown in Figure 2, was investigated and analyzed following the operating parameters: initial enantiomeric excess (ee_o_), residence time, and suspension wt %. In all experiments, the concentration of DBU, the initial solid density of the suspension, and the wt % of crystals dissolved at *T*_2_ were kept constant. By fixing *T*_1_ and keeping Δ*T* constant, the amount of dissolved suspension was determined at *T*_2_. The TCCB experiments and the operating parameters selected are given in Table 2.

Initial tests, TCCB 01–02, were done for testing continuous suspension transfer. This was performed after calibrating the pumps to avoid shifting of suspension volume in vessels. Samples were taken after every hour and were analyzed via Chiral HPLC. Continuous flow and transfer between the two vessels caused a very slow increase in ee% with respect to process time. The reason for this slow evolution could be due to continuous transfer resulting in a very low residence time of the suspension in the vessel, thus allowing insufficient time for crystals to grow in the saturated solution. The volume in the cold vessel was kept slightly higher than that in the hot vessel in order to increase the residence time for the crystals to grow further. Still, this strategy was not effective and leaving it overnight resulted in only a 4 ee% increase within 24 h duration. Visual inspection showed that the inlet tubes were clogged by agglomerated crystals, and as a result, the remaining crystals were fines or were completely dissolved in both vessels. Progression of the process was relatively too slow; hence, the experiment was stopped. To minimize the issue of tube clogging, TCCB-02 was carried out with a reduced suspension density of 0.5%. A similar trend was observed with no or similar ee% evolution and all crystals dissolved in *V*_2_ after clogging occurred overnight. For the initial TCCB (01 and 02) during continuous transfer between the two vessels, the process generated very slow increase in ee%, which increased with respect to process time because the continuous transfer resulted in a too low residence time in both vessels. In both experiments, for prolonged observation after leaving overnight and due to higher settling velocities of the crystals, continuous flow resulted in clogging of tubes. This suggested that continuous pumping might not be suitable for this compound and a slower flow rate prevented pumping of suspension crystal in the tubes. An alternative pumping scheme was proposed to further investigate the two-vessel system. The main aim of this pumping profile was to increase the residence time to allow the kinetics of the process to occur in the vessel.

### Effect of the Modified Pulsating Pumping Profile on the Two-Vessel System

For these experiments, a modification was made to the pumping profile by including pulsating flow and reverse flow (emptying the tubes, no flow) for increased residence time of the suspension in both vessels.

**Figure 5:**
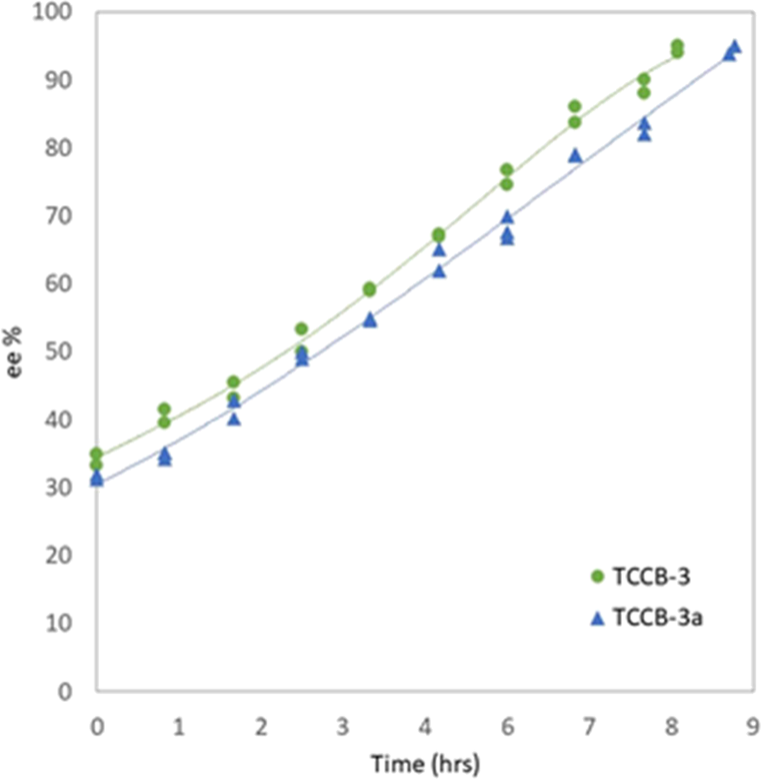
Evolution of ee% as a function of time in coupled vessel experiments for TCCB-03 and 03a: green circles and blue triangles represent the data points of the two repetitions with similar initial conditions. All other experiments were conducted at least two times (data points shown as average).

Experiment TCCB03/03a, Figure 5, was successfully performed with the new flow scheme as it was successful in transferring a limited volume between the vessels (20% of vessel volume) at each pump cycle profile shown in Figure 2. With a regular interval and reverse flow, this eliminated the issue of blocking the inlet tubes. The effect on vessel temperature during suspension transfer with the pulsating flow mechanism was *T*_1_ = 25 °C ± 0.5 °C and *T*_2_ = 34 °C ± 1.2 °C.

For 30% initial excess (ee_o_), complete deracemization in TCCB-03 and 03a was achieved using the coupled batch technique in approximately 9 h utilizing the programmed pump. Visual inspection after the experiment showed no clogging in the inlet or outlet tubes. Mixtures of different NMPA batches were used for the initial ee_o_ as a test. Increased residence time by maintaining no flow time enabled the process to reach close to 95 ee% within 9 h, as shown in Figure 5: the first experiment that reached complete deracemization.

### Effect of the Initial Enantiomeric Excess

**Figure 6:**
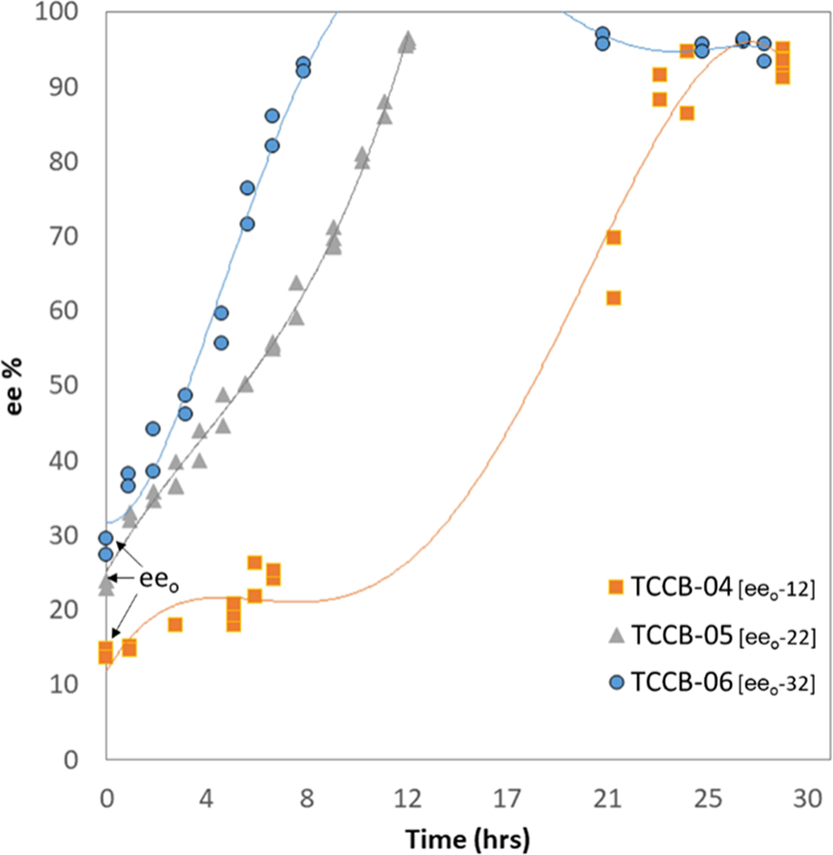
Evolution of the enantiomeric excess ee% vs time in coupled vessel experiments at different initial enantiomeric excess ee_o_. The results are represented by orange squares (TCCB-04, ee_o_ = 12%), gray triangles (TCCB-05, ee_o_ = 22%), and blue circles (TCCB-06, ee_o_ = 32%). Total solution volume *V* = 150 mL (*V*_1_ + *V*_2_).

In recent studies, as mentioned in the Introduction, single-vessel attrition-enhanced temperature cycling Viedma ripening experiments and model studies showed that deracemization becomes faster with a larger initial enantiomeric excess eeo.^9,26^ To understand whether a similar effect holds, the two-vessel setup experiments with ee_o_ of 12, 22, and 32% were performed. All other parameters were kept constant and operating conditions were kept the same. The results are shown in Figure 6.

Experiment TCCB-04 was performed with the lowest ee_o_ (12%) and took around 24 h to attain deracemization *>*95%, TCCB-05 took 13 h with an ee_o_ of 22% and TCCB-06 ee_o_ (32%) completed within 9 h duration. Samples were taken every hour, and the trend line indicated that it must follow a faster rate with higher initial enantiomeric excess. Experiments TCCB-3 and 3a shown in Figure 5, conducted at similar conditions to TCCB-06, showed the end of deracemization after 9 h and gave a similar trend line. These results indicated that an increase in initial *ee*_o_ from 12 through to 32% causes more rapid deracemization evolution. With 12% ee_o_, the slow upward shift of slope was observed until it reached 20 ee% and it remained flattened for several hours. Next day sampling analysis of the suspension showed that it jumped to 71% after 20 h, resulting in an overnight sharp increase in ee evolution and then reached *>*95% around 24 h duration. This initial flattening or slow evolution was not observed with higher ee_o_ experiments that resulted in a much faster evolution and reaching the final point. To explain this effect, higher ee_o_ results in a greater number of crystals in the suspension (more crystal sites, higher surface area), and as a result, faster dissolution and resolution in the liquid phase in the hot vessel leads to faster resolution to form the enriched enantiomer.

### Effect of Variation in Residence Time

**Figure 7:**
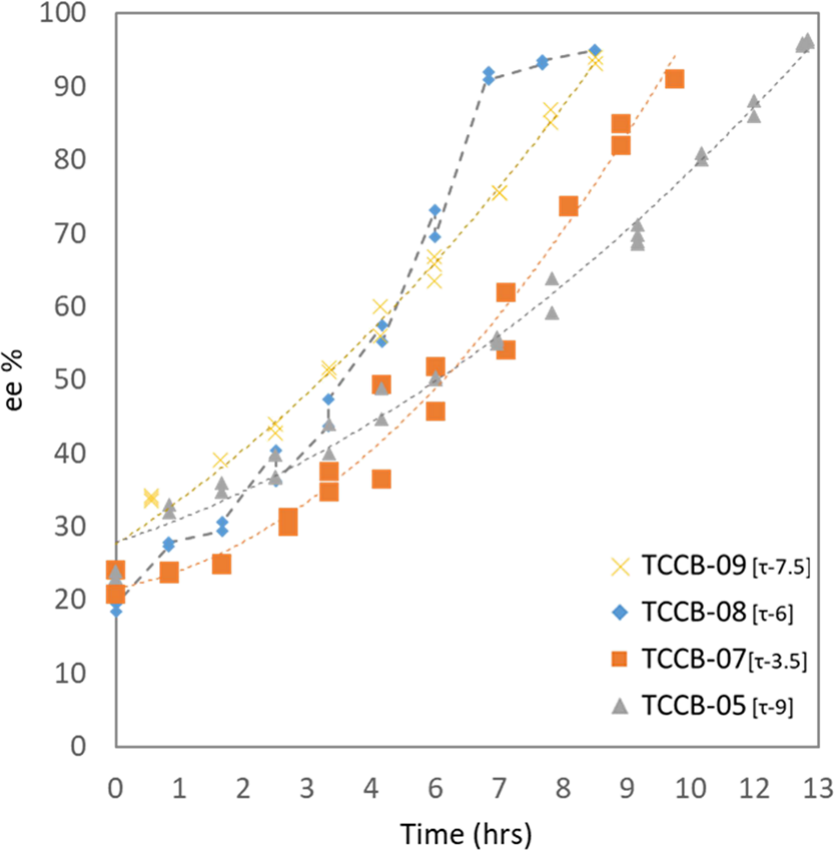
Evolution of ee at varied residence time in coupled vessel experiments TCCB-05 to 09. Residence time variation (3.5, 6, 7.5, and 9 min) is kept the same for both vessels τ_1_ and τ_2_ = τ to analyze its effect on overall deracemization process progress.

The effect of varying the residence time in each vessel with an increase in the no-flow pumping profile stage on ee was investigated. These experiments were performed by keeping all other parameters constant (Δ*T* = 9 °C, eeo = 22–24%). Reducing the no flow time from 5 to 3 min caused a reduction in residence time to 7.5 min in both vessels. Residence times of (τ1 and τ2) 3.5, 6, 7.5, and 9 min were examined, and the results are shown in Figure 7.

The result for TCCB-05 showed that a residence time τ of 9 min enabled the ee evolution to reach 95 ee % within 13 h duration and reducing τ to 7.5 and 6 min in TCCB-09 and 08 further reduced the duration to 9 and 7.8 h, respectively. For all these experiments the ee_o_ was kept the same. However, reducing the overall residence time to 3.5 min resulted in a slower deracemization duration to over 10 h to achieve complete deracemization for TCCB-07. At low mean residence times in the two vessels, a fluid element experienced (on average) a more rapid change in temperature. Reducing the residence time to 3 min increased the rate of deracemization, resulting in faster evolution of ee, as shown in Figure 7. Although the upward trend of the slopes was similar, this showed that there was a limit to residence time in the vessel and reducing it further would not increase the evolution of deracemization. This might be due to growth rate limitation and reducing the residence time limits for the crystal to grow and re-dissolve more in slower evolution. This was also observed in the continuous flow pumping profile where the residence time was further reduced to τ1 and τ2 to 2.67 and 1.3 that resulted in very slow deracemization progression.

### Effect of Variation in Suspension Density

**Figure 8:**
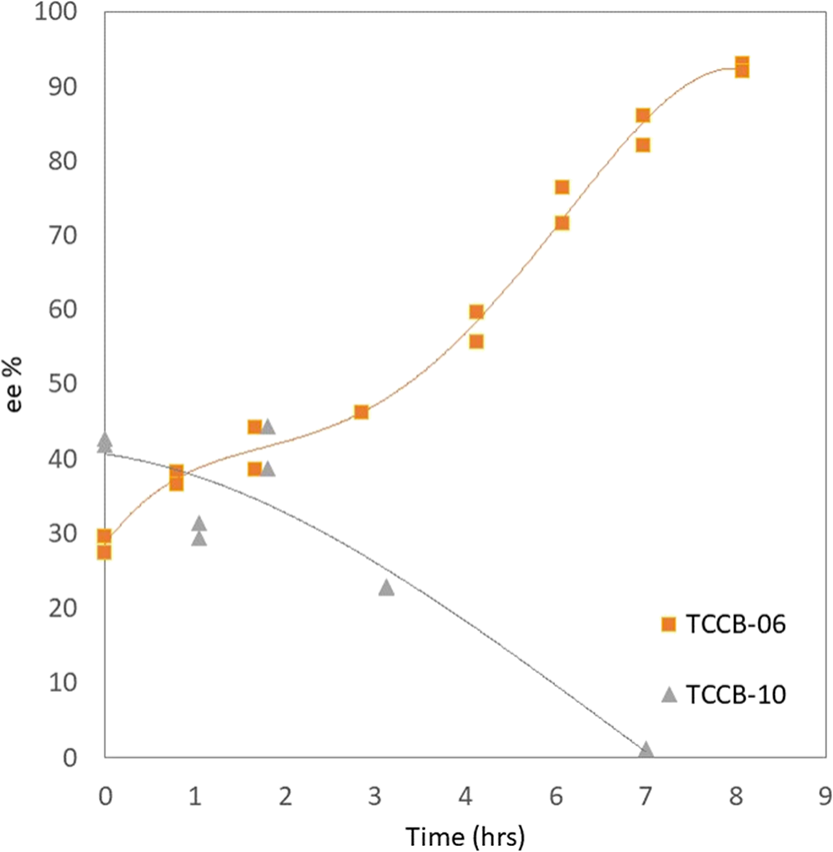
Evolution of the ee vs time in coupled vessel experiments: Effect of variation in suspension wt %. Orange squares (TCCB-06 with 1.5 wt %) and gray triangles (TCCB-10 with 0.5 wt %).

The TCCB-10 experiment was performed at 0.5 wt %, and the details are given in Table 2. The aim of reducing the suspension density was to investigate for a full dissolution test (complete dissolution of suspension in the hot vessel) in which almost all the crystals were dissolved at 34 °C in vessel 2 (0.0132 g was left undissolved). However, vessel 1 was kept at a temperature of 25 °C to maintain the supersaturation for crystal growth. HPLC results of experiment TCCB-10 are shown in Figure 8 and are compared with TCCB-06 with 1.5 wt % suspension density and similar initial conditions. Increasing the dissolved amount was achieved through varying the hot vessel temperature or lowering the suspension density. However, there were certain limitations as lower wt.% can result in secondary nucleation of the unwanted enantiomer. HPLC analysis indicated that instead of an increase in ee%, there was a significant drop in enantiomeric excess, even starting from a high initial excess of 40% ee_o_. Due to the incoming hot solution stream into the cold vessel during mixed suspension transfer between the two vessels, more crystals were being dissolved in the liquid phase of both vessels, resulting in more fine crystals. At 7 h interval sampling analysis of TCCB-10, it reached near 0 ee%, which indicates that the existing suspension has become a racemic mixture. Afterward, it was challenging to have a suitable sample for analysis as extremely fine size crystals were obtained in both vessels and it was not possible to filter them so that the reaction was stopped at this stage.

As a result, very low suspension selection was not a suitable option for this system configuration, as full dissolution of the suspension in the vessel resulted in racemization of enantiomers back to the racemic form so it showed that the reaction reversed regardless of the 40% initial excess of one enantiomer. In order to sustain the partially dissolved suspension in both vessels, it was recommended to increase the initial suspension density in such a way that only 30–60% dissolved in the higher temperature vessel. A suitable suspension density and Δ*T* were selected to ensure that no complete dissolution occurred in the vessel at higher temperatures.

## Conclusions

The TCCB confirmed an improved alternative setup for deracemization via temperature cycling. These experiments showed successful resolution of an NMPA model compound and its capability of reaching high purity in a shorter process time as compared to a single vessel setup. Initial operating parameters were investigated by performing single vessel experiments via Crystal16 equipment. The issue of clogging of the transfer lines was resolved by the modified pumping profile that enabled running the process for longer durations and successfully obtaining reproducible results. Results showed that this approach was a more effective process in terms of faster completion to >95 ee% within 8–9 h and all tests were examined at higher volume levels (150 mL). Full conversion (ee = 100%) was not reached at the end of the process, which could be due to the fact that the larger crystals grown were not sampled into the hot vessel and as a result were not converted into the desired enantiomer. A dispersing tool to break the agglomerated crystals could be employed to resolve this issue.

The effect of initial ee variation showed a similar behavior as compared to the single vessel experiment performed in previous studies, where higher initial excess (ee_o_) led to faster deracemization. However, selection of ee_o_ should be done in a way that prevents sacrificing process productivity (a larger amount of product was utilized to initiate the next experiment, thus reducing the overall yield of process). The residence time of the vessels was a critical parameter and must be optimized to allow a large number of cycles to take place as it drives the rate of enantiomeric conversion occurring in the hot vessel. At low mean residence times in both vessels, the suspension experiences (on average) a more rapid change in temperature. However, the residence time of the system must also be long enough to allow sufficient growth of the desired enantiomer, without the cycle length having a negative impact on the overall process time. Similarly, slow ee evolution was observed during the continuous cycling mode as a result of the low residence time in both vessels. The optimized residence time for NMPA was obtained as approximately 6 min. A hot vessel full dissolution test is not recommended as dissolution becomes dominant, as it resulted in racemization of the suspension back to its racemic mixture.

Utilization of the two-vessel system proved to be more effective in terms of time and energy required to maintain the vessel temperatures, as both were at different fixed temperatures, and negligible temperature fluctuations were involved. However, single vessel temperature cycling experiments required more time (rate limited) and faster cooling/heating power in terms of performing temperature variation for temperature cycles. Designing the two-vessel system requires precise analysis of reaction kinetics of the system and the optimized programmed pumping mechanism (shown in Figure 2) that enables precise suspension transfer, and selection of optimized parameters was critical for efficient performance of the overall process. In further work, the impact of additional variables such as the temperature swing between the two vessels and higher suspension densities on the process should be investigated with the intention of finding the optimum process conditions. Optimization steps could be utilized to develop the process for a range of chemical systems at industrial scales. In terms of process scale-up, it could be conveniently implemented due to its simple setup and low cost. Results showed that complete deracemization was achieved faster in this process as compared to one-vessel temperature cycling (Crystal16 Experiments) or attrition-enhanced process (involvement of beads for grinding). As compared to one-vessel, a large quantity of suspension at an industrial scale required more process time to achieve and maintain heating and cooling temperatures cycles, and due to this requirement, higher heat transfer areas would require more power, hence more cost. Hence, we can conclude that the modified coupled vessel system proposed initially by Suwannasang proves to be more efficient as it allows faster resolution with comparison of attrition-enhanced Viedma ripening and single vessel temperature cycling. It requires shorter cycle times to achieve complete deracemization and overall can be one of the most promising candidates for scale-up since it is a one-step operation and more economically viable to maintain two vessels at fixed temperatures.
